# Clinical assessment of children with renal abscesses presenting to the pediatric emergency department

**DOI:** 10.1186/s12887-016-0732-5

**Published:** 2016-11-22

**Authors:** Chun-Yu Chen, Huang-Tsung Kuo, Yu-Jun Chang, Kang-Hsi Wu, Wen-Chieh Yang, Han-Ping Wu

**Affiliations:** 1 Division of Emergency Medicine, Department of Pediatrics, Changhua Christian Hospital, Changhua, Taiwan; 2 School of Medicine, Chung Shan Medical University, Taichung, Taiwan; 3 School of Medicine, Kaohsiung Medical University, Kaohsiung, Taiwan; 4 School of Medicine, China Medical University, Taichung, Taiwan; 5 Department of Developmental and Behavioral Pediatrics, Children’s Hospital of China Medical University, Taichung, Taiwan; 6 Laboratory of Epidemiology and Biostastics, Changhua Christian Hospital, Changhua, Taiwan; 7 School of Post-Baccalaureate Chinese Medicine, College of Chinese Medicine, China Medical University, Taichung, Taiwan; 8 Department of Hemato-Oncology, Children’s Hospital, China Medical University Hospital, China Medical University, Taichung, Taiwan; 9 Division of Pediatric General Medicine, Department of Pediatrics, Chang Gung Memorial Hospital at Linko, No. 5, Fu-Hsin Street, Kweishan, Taoyuan Taiwan; 10 College of Medicine, Chang Gung University, Taoyuan, Taiwan

**Keywords:** Renal abscesses, Children, Emergency department

## Abstract

**Background:**

Renal abscesses are relatively uncommon in children but may result in prolonged hospital stays and life-threatening events. We undertook this study to analyze the clinical spectrum of renal abscesses in children admitted to the pediatric emergency department (ED) and to find helpful clinical characteristics that can potentially aid emergency physicians for detecting renal abscesses in children earlier.

**Methods:**

From 2004 to 2011, we retrospectively analyzed 17 patients, aged 18 years or younger, with a definite diagnosis of renal abscess admitted to the ED. The following clinical information was studied: demographics, clinical presentation, laboratory testing, microbiology, imaging studies, treatment modalities, complications, and long-term outcomes. We analyzed these variables among other potential predisposing factors.

**Results:**

During the 8-year study period, 17 patients (7 males and 10 females; mean age, 6.1 ± 4.5 years) were diagnosed with renal abscesses on the basis of ultrasonography and computed tomography findings. The 2 most common presenting symptoms were fever and flank pain (100% and 70.6%, respectively). All of the patients presented with leukocytosis and elevated C-reactive protein (CRP) levels. Organisms cultured from urine or from the abscess were identified in 11 (64.7%) patients, and *Escherichia coli* was the most common organism cultured. All patients were treated with broad-spectrum intravenous antibiotics with the exception of 4 children who also required additional percutaneous drainage of the abscess.

**Conclusions:**

Renal abscesses are relatively rare in children. We suggest that primary care physicians should keep this disease in mind especially when children present with triad symptoms (fever, nausea/vomiting, and flank pain), pyuria, significant leukocytosis, and elevated CRP levels. However, aggressive percutaneous drainage may not need to be routinely performed in children with renal abscesses.

## Background

Renal abscesses are relatively uncommon in children but may result in a prolonged antibiotic course, increased length of hospital stay, high treatment cost, or life-threatening complications [1–4]. The clinical presentation of renal abscess may be nonspecific, and can include fever, nausea/vomiting, flank pain, abdominal pain, elevated erythrocyte sedimentation rate, leukocytosis, and positive blood/urine cultures [3–5]. Early diagnosis is imperative to minimize the potential for prolonged admission, high treatment costs, and life-threatening complications. However, it may be challenging for emergency physicians to make an early diagnosis of patients with renal abscesses based on the clinical presentation at the time of their emergency department (ED) visits.

With the progressive improvement of ultrasonography (US) and computed tomography (CT), a definitive diagnosis of renal abscess may be made more readily than was previously possible [4, 6]. However, renal abscesses may be difficult to distinguish from certain renal lesions on US or some renal diseases based on clinical assessment, such as acute pyelonephritis, acute lobar nephronia, and renal mass [7]. CT shows specific findings in cases of renal abscess and it remains the best choice for diagnosis. In addition, CT has the advantage of providing a distinction between renal and perirenal abscesses [3, 8–10]. In this study, we analyzed the clinical spectrum of renal abscesses in patients who presented to the pediatric ED with the goal of finding initial clinical characteristics that can help emergency physicians diagnose renal abscess earlier and to improve the prognosis for patients with this disease.

## Methods

This is a retrospective study of pediatric patients aged 18 years or younger who presented to the ED with a discharge diagnosis of renal abscess from January 2004 to December 2011. The study was approved by the Institutional Review Board of the Changhua Christian Hospital, and the necessity to obtain written consent was waived because of its retrospective nature.

We identified 36 potentially eligible patient visits by searching the Changhua Christian Hospital health records database using the following search term in the primary or secondary discharge diagnosis fields: renal and perinephric abscess (*ICD-9* 590.2). We excluded patients with (1) perinephric abscess on US or CT, (2) age >18 years, and (3) no ED presentation prior to hospitalization. In total, 17 patients were included in this series.

The following information was obtained from the medical records of each patient: age, gender, clinical symptoms and signs (such as fever, abdominal pain, diarrhea, nausea/vomiting, and flank pain), laboratory tests [white blood cell (WBC) counts, hemoglobin (Hb) level, platelet counts, blood urea nitrogen (BUN), creatinine (Cr), C-reactive protein (CRP) levels], microbiology, imaging findings, treatment modalities, complications, and long-term outcomes. We also evaluated the follow-up data, including imaging findings and any documentation of renal sequelae.

### Statistical analysis

Data of categorical variables were analyzed by the chi-square test or Fisher’s exact test when appropriate. Continuous variables were analyzed by the Mann–Whitney *U* Test and the Kruskal–Wallis Test. A *P* value of <0.05 was considered to be statistically significant. Distributions of variables were reported as percentages and means ± standard deviation (SD). Statistical analyses were performed with SPSS software (version 15.0, SPSS, Inc., Chicago, IL, USA).

## Results

### Demographics and clinical presentations

During the 8-year study period, 17 patients (7 males and 10 females; mean age, 6.1 ± 4.5 years; range, 6 months to 15 years) who presented to the ED with renal abscesses were enrolled. In this study, 11 (64.7%) of the 17 children were aged <6 years. The 2 most common symptoms at presentation were fever and flank pain (100% and 70.6%, respectively), and 7 patients (41.2%) presented with fever for >7 days before diagnosis (Table 1). Children with a single abscess had a shorter duration of fever before admission (4.4 ± 3.31 vs. 6.29 ± 4.63 days, respectively) and presented with a lower anorexia or vomiting percentage (30% vs. 71.4%, respectively). Three children had right grade III–V vesicoureteral reflux (VUR) and 1 patient had type 2 diabetes mellitus as a comorbidity.

**Table 1:** Demographics and clinical presentations of patients with renal abscesses

Variables			Age (years)				
	Total (*n* = 17)		0 to 6 (*n* = 11)		7 to 18 (*n* = 6)		*P* value
	n	%	n	%	n	%	
Gender							
Female	10	58.8	5	45.5	5	83.3	0.304
Male	7	41.2	6	54.5	1	16.7	
Fever	17	100.0	11	100.0	6	100.0	
Prolong fever >7 days (before diagnosis)	7	41.2	6	54.5	1	16.7	0.304
Abdominal pain	4	23.5	1	9.1	3	50.0	0.099
Diarrhea	1	5.9	0	0.0	1	16.7	0.353
Nausea or vomiting	8	47.1	5	45.5	3	50.0	1.000
Flank pain	12	70.6	6	54.5	6	100.0	0.102
Admission unit							
Ward	16	94.1	11	100.0	5	83.3	0.353
ICU	1	5.9	0	0.0	1	16.7	

*ICU*intensive care unit

### Laboratory findings and microbiological examination

Of the 17 patients with renal abscesses, all presented with leukocytosis and elevated CRP level (normal range ≥8 mg/L). The mean WBC count was 23,070 ± 10,576/μL (range 11,300–55,500/μL) (Table 2). Thirteen patients (76.5%) had a WBC count of >15,000/μL and 10 children (58.8%) presented with a left shift. The CRP levels were significantly higher in the adolescent group (*P* = 0.027) and the mean was 164 ± 113 mg/L (range, 11 to 355 mg/L). However, the platelet count was significantly higher in children aged ≤6 years (*P* = 0.003). Ten patients (58.8%) had pyuria [>5 leukocytes per high-power field (HPF)]; 6 of these 10 patients also presented with hematuria (>5 RBCS per HPF). Moreover, children who presented with a single abscess tended to have lower pyuria and urine culture positivity than those who presented with multiple renal abscesses (50% vs. 71.4%; 30% vs. 71.4%, respectively).

**Table 2:** Comparison of laboratory tests of patients with renal abscesses based in different age groups

Age (years)		0 to 6			7 to 18			Total		
Laboratory data	N	Mean	SD	N	Mean	SD	N	Mean	SD	*P* value
WBC (× 10^9^/L)	11	23902.73	11602.34	6	21545.00	9188.50	17	23070.59	10576.71	0.421
Hb (mg/dl)	11	11.26	1.06	6	11.95	1.26	17	11.51	1.15	0.266
Platelet count (× 10^9^/L)	11	470.55	166.82	6	221.00	56.37	17	382.47	183.02	0.003^a^
CRP (mg/L)	11	116.8	80.2	6	25.14	11.93	17	164.3	113.4	0.027^a^
BUN (mg/dl)	5	9.62	8.41	3	11.33	7.09	8	10.26	7.46	0.655
Creatinine (mg/dl)	6	0.58	0.21	3	0.78	0.03	9	0.65	0.20	0.120

*WBC* white blood count, *Hb* hemoglobin, *CRP* C-reactive protein, *BUN* blood urea nitrogen

^a^Statistically significant by Kruskal–Wallis Test

Of the 17 cases, organisms cultured from blood, urine, or abscess were identified in 11 (64.7%) patients (Table 3). *Escherichia coli* was the most common organism cultured from both abscesses and urine samples. Moreover, *KlebsieIla pneumoniae* was cultured in only 1 urine sample, and oxacillin-susceptible *Staphylococcus aureus* was recovered in 1 of 4 abscess culture samples. Blood cultures were negative in all patients.

**Table 3:** Microbiological results of 17 children with renal abscesses

Microbial findings	Patients no. (%)
Urine culture (*n* = 17)	
Negative	9 (52.9)
*Escherichia coli*	7 (41.2)
*Klebsiella pneumoniae*	1 (5.9)
Abscess culture (*n* = 4)	
Negative	1 (25)
*Escherichia coli*	2 (50)
*Staphylococcus aureus*	1 (25)
Blood culture (*n* = 17)	
Negative	17 (100)

### Imaging findings and management

All patients were diagnosed with renal abscesses by imaging (CT or US). In 12 (70.6%) patients, the abscesses were confined to the right kidney (Table 4). The average abscess size was 27.8 ± 9.3 mm and more than half of the children had a single abscess on imaging studies.

**Table 4:** Imaging results of 17 children with renal abscesses

Imaging findings	Patients no. (%)
Side	
Right	12 (70.6)
Left	3 (17.6)
Bilateral	2 (11.8)
Number	
Single	10 (58.9)
Multiple	7 (41.1)
Size	
< 1 cm	0
1to 3 cm	10 (58.9)
> 3 cm	7 (41.1)

Multiple, >1 abscess or multilobulated

All patients were treated with broad-spectrum intravenous antibiotics for a mean duration of 16.6 ± 8.8 days. Four (23.5%) of the 17 patients had a combination of broad-spectrum intravenous antibiotics therapy and percutaneous abscess drainage by US-guide needle aspiration. The mean duration of hospitalization in patients who received percutaneous abscess drainage was 24.3 ± 12.5 days vs. 15.5 ± 7.3 days for patients receiving antibiotics alone. All of the 17 patients recovered completely and none required an open drainage or nephrectomy. During the follow-up examinations, we did not find any abscess recurrence in these patients.

## Discussion

Renal abscess is a rare clinical condition in children, and the prevalence in children is still unknown. During our 8-year study period, of approximately 200,000 children presenting to our pediatric ED, only 17 pediatric patients were discharged with the final diagnosis of renal abscess. However, renal abscess is one of the most severe forms of renal parenchymal infection in children and may lead to renal loss and even death. The female to male ratio of pediatric renal abscess in our study was 1.4:1. This result is similar to the largest study of pediatric renal abscess in which the female to male ratio was 1.7:1 [4]. As previously reported, the most common predisposing risk factors of renal abscesses in adults are diabetes mellitus, nephrolithiasis, and ureteral obstruction [11, 12]. In the pediatric population, urological abnormality (VUR, ureteropelvic junction obstructions, and calyceal diverticulum) and urolithiasis seem to be the most important predisposing risk factors for renal abscesses [3, 13–15]. In our series, a 15-year-old girl had diabetes mellitus, and 3 children presented with VUR. Thus, preexisting structural abnormities of the urinary tract and systemic diseases are not necessarily prerequisites for renal abscess formation.

Clinical diagnosis of renal abscess is often difficult because the symptoms are often nonspecific, especially in younger children. In 2008, Cheng et al. observed fever in 100% of patients, nausea and vomiting in 44.4%, abdominal pain in 35.6%, and flank pain in 31.1% [4]. Of the 17 patients in our study, fever was also observed in all of them (100%), and 41.2% presented with prolonged fever for >7 days prior to diagnosis. The mean fever duration before admission of these 17 children was 5.18 ± 3.89 days, and children who were aged <6 years had longer fever durations before admission (6.27 ± 4.31 days). This indicates that younger children who present with renal abscesses usually show less typical symptoms or signs, which may lead to delay diagnosis and treatment; in our study, 41.2% of the younger children presented with the triad of fever, nausea and vomiting, and flank pain.

Laboratory tests are not specific for the diagnosis of renal abscess. In our study, all 17 patients had leukocytosis with elevated CRP levels, and 64.7% of them presented with WBC counts >15,000/μL. In addition, 58.8% of the 17 children showed pyuria, but none manifested renal function impairment. According to these results, we suggest that emergency physicians should keep the diagnosis of renal abscess in mind when children present with triad symptoms, pyuria, and significant elevated serum WBC counts and CRP levels.

We compared the clinical presentations and laboratory results between the children who presented with multiple abscesses and single abscess. We found that flank pain, anorexia or vomiting, significant leukocytosis, pyuria, and urine culture were more frequently noted in the multiple-abscess patient group. Early imaging studies and intravenous antibiotics are advocated in this group of children to minimize hospital stay.

According to previous studies, renal abscess may result from hematogenous spread or ascending infections due to reflux or stasis of infected urine, and the most common pathogens isolated in children are *E. coli* and *S. aureus* [3, 4, 16]. In adults, the most frequent microorganisms yielded on culture are *E. coli* and *K. pneumoniae* [11, 12]. In addition, anaerobic bacteria have also been reported to play an important role in pediatric renal abscesses [17]. In our series, *E. coli* was not only the leading cultured organism of renal abscesses but also the most common pathogen isolated from urine. However, all blood cultures were negative in the current study. These results may indicate that ascending infections from the urinary tract may play a more important role than hematogenous spread in the development of renal abscesses in children.

Delays in diagnosis of renal abscesses may result in increased morbidity and potential mortality. However, the diagnosis is not easy to make without US or CT studies. US is particularly useful in the diagnosis of pediatric renal abscess [4, 18]. However, CT has greater sensitivity and the best diagnostic accuracy for these lesions [4, 6, 7]. In addition, many experts still suggest that even when renal abscesses are identified by US, CT with contrast enhancement should be performed to distinguish between renal and perirenal abscesses [2–5]. In our study, all patients had undergone US and CT, and, in 12 (70.1%) children, renal abscesses were initially diagnosed by US. We suggest that US can be used as a screen and follow-up tool, and CT can be used to confirm the diagnosis of renal abscesses in children as the initial US does not always provide a definitive diagnosis. In our series, 12 of 17 patients (70.6%) had renal abscesses of the right kidney. However, we did not find the same results in previous larger studies in children or in adults. Because of the relatively small sample size, we cannot make the conclusion about the lateralization of renal abscess in our study.

To prevent life-threatening complications caused by renal abscesses, early recognition and appropriate treatment is important. Abscess formation is more likely under conditions of impaired immunity [19, 20]. According to previous studies, appropriate antibiotic coverage combined with percutaneous or open surgical drainage could dramatically decrease morbidity and mortality from this infection [3, 11, 21, 22]. However, in an adult study, all abscesses <3 cm in diameter resolved with 4–6 weeks of antibiotic therapy alone, and percutaneous drainage was as effective as open surgery for large and medium-sized abscesses [23]. In a recently published larger pediatric study, only 3 of 45 patients required CT-guided percutaneous drainage in addition to antibiotic management, and none had to undergo open drainage or nephrectomy [4]. In our report, 13 of 17 children were treated successfully with antibiotics alone; 4 children required US-guided percutaneous drainage in addition to antibiotic management, and none needed open drainage or nephrectomy. This supports initial management of pediatric renal abscess with a prolonged antibiotic course and US monitoring, reserving percutaneous drainage for larger renal abscesses or cases refractory to antibiotic therapy. However, definite treatment guidelines for percutaneous drainage in managing children with renal abscesses requires further, larger prospective studies.

The present study has a number of limitations. First, in a retrospective single-center review of medical records, some details of history and physical examinations may not be rigorously documented. Second, the relatively small sample size may fail to address definite factors of early diagnosis and the best treatment modality of renal abscess in children. Finally, the voiding cystourethrogram was not routinely performed in all patients, and the definite role of VUR in renal abscess was therefore difficult to address. These limitations may have led to bias in analyzing the clinical spectrum of renal abscesses in children who presented to the ED.

## Conclusions

Renal abscesses are relatively uncommon in children but may lead to prolonged admission and severe complications. Primary physicians should keep the diagnosis of renal abscess in mind when children present with triad symptoms (fever, nausea/vomiting, and flank pain), pyuria, and significantly elevated serum WBC count (>15,000/μL) and CRP level (>50 mg/L). Children with renal abscesses usually have good outcomes with early diagnosis and institution of broad-spectrum antibiotic therapy. Finally, percutaneous drainage should not be routinely required in children with renal abscesses.

## Abbreviations

BUN: Blood urea nitrogen
Cr: Creatinine
CRP: C - reactive protein
CT: Computed tomography
*E. coli*: *Escherichia coli*
ED: Emergency department
Hb: Hemoglobin
HPF: High power field
ICU: Intensive care unit
*K. pneumoniae*: *KlebsieIla pneumoniae*
*S. aureus*: *Staphylococcus aureus*
SD: Standard deviation
US: Ultrasonography
VUR: Vesicoureteral reflux
WBC: White blood cell
